# Lower Bolting Height of Winter Rapeseed (*Brassica napus* L.) Enhances Cold Stress Tolerance and Adaptability to Arid–Frigid Regions in Northern China

**DOI:** 10.3390/plants15091378

**Published:** 2026-04-30

**Authors:** Zhuanhong Liang, Sheng Chen, Tingting Fan, Wenxin Yang, Jianzhong Sang, Junyan Wu, Li Ma, Yuanyuan Pu, Wangtian Wang, Lijun Liu, Haiqing Liu, Gang Yang, Wancang Sun

**Affiliations:** 1 College of Agronomy, Gansu Agricultural University, Lanzhou 730070, China; 107332202250@st.gsau.edu.cn (Z.L.);; 2 The UWA Institute of Agriculture, The University of Western Australia, Perth 6009, WA, Australia; 3 State Key Laboratory of Aridland Crop Science, Gansu Agricultural University, Lanzhou 730070, China; 4 College of Life Science and Technology, Gansu Agricultural University, Lanzhou 730070, China; 5 School of Agriculture and Bioengineering, Longdong University, Qingyang 745000, China

**Keywords:** *Brassica napus* winter rapeseed, dry–cold region, bolting height, cold tolerance

## Abstract

Bolting height is a key genetic trait that affects the stress tolerance, environmental adaptation, and winter survival of *Brassica napus* winter rapeseed. It is particularly important for enhancing winter survival in the arid–frigid regions. This study aimed to elucidate the genetic relationship between bolting height and cold stress tolerance, thereby supporting breeding for enhanced cold tolerance. Ninety-five winter rapeseed accessions were used in this study. Through both spring and autumn sowing trials, the dynamic changes in bolting height under different environments were systematically analyzed, and the genetic stability of bolting height as well as its correlation with cold tolerance were clarified. Bolting height showed consistent variation trends between spring and autumn sowing trials, exhibiting high genetic stability. It displayed an extremely significant negative correlation with cold tolerance: genotypes with lower bolting height possessed stronger cold tolerance. The regulatory mechanism underlying low bolting and cold tolerance was revealed at cellular and molecular levels. Low bolting accessions exhibited flat and broad shoot apical meristems, with small and compact cells, a high nucleoplasmic ratio, and indistinct vacuoles. The gibberellin synthesis gene *BnaA06g24070D* was downregulated, while the key cold-tolerant gene *BnCBF5* was upregulated. Exogenous hormone treatment preliminarily verified the causal regulatory effect of bolting height on cold tolerance. In both spring and autumn sowing trials, bolting height at the initial flowering stage showed an extremely significant positive correlation with vernalization index, with correlation coefficients of 0.80 and 0.78, respectively. Lower bolting height corresponded to a smaller vernalization index and stronger temperature sensitivity. Moreover, bolting height at the initial flowering stage showed an extremely significant negative correlation with comprehensive cold tolerance scores, with correlation coefficients of −0.77 and −0.80, respectively. Low-bolt materials had significantly higher overwintering rates and comprehensive cold tolerance scores, as well as a markedly lower semi-lethal temperature (LT_50_), compared with high-bolt accessions. Low-bolt accessions presented significantly prolonged bolting stage, bud stage, initial flowering stage, and whole growth durations, and their agronomic trait stability across years was significantly superior to that of high-bolt accessions. This study confirmed that low bolting height is a crucial breeding trait for the cold tolerance of winter rapeseed, and thus an important selection indicator for the cold tolerance improvement of winter rapeseed in arid–frigid regions in northern China.

## 1. Introduction

*Brassica napus* L. accounts for more than 90% of the total rapeseed planting area in China, with an average yield of 2.079 t/ha. It possesses comprehensive advantages, including lodging resistance, excellent agronomic traits, and superior quality, and serves as the dominant rapeseed variety in domestic rapeseed production [1,2]. According to sowing season, rapeseed can be divided into spring rapeseed and winter rapeseed. Traditionally, the planting areas of *B. napus* winter rapeseed have long been concentrated in the Yangtze River Basin and regions south of 35° N latitude, accounting for approximately 80% of China’s total rapeseed planting area [3,4,5]. In recent years, the “northward expansion of winter rapeseed” initiative has highlighted the dry and cold regions in northern China as a key area for industrial development. These regions possess abundant light and thermal resources, with significant potential for arable land expansion, playing a crucial role in national grain and oil security [6]. However, these regions experience long winter periods with extremely low temperatures (below −20 °C) and limited precipitation. Insufficient cold stress tolerance poses a major challenge to overwintering survival, so improving cold stress tolerance in winter rapeseed has been a long-standing research priority [7].

Cold stress tolerance is a complex quantitative trait controlled by multiple genes [8,9]. Plants respond to low-temperature stress by accumulating osmoprotectants (e.g., proline and soluble sugars) and enhancing the activity of antioxidant enzymes such as superoxide dismutase and peroxidase. These mechanisms help maintain cellular homeostasis [10]. Cold-responsive genes, including *BnCBF*, *BnCOR*, and *BnICE*, regulate signaling pathways to improve cold tolerance [11,12]. Furthermore, genome-wide association studies and quantitative trait locus (QTL) mapping have identified numerous loci and candidate genes associated with low-temperature tolerance [13]. The botanical traits of winter rapeseed in northern China, particularly premature bolting, are closely linked to cold stress tolerance. Premature bolting elevates the shoot apical meristem, increasing its exposure to freezing injury and reducing overwintering survival [14]. Li et al. reported a significant correlation between meristem height and cold stress tolerance, where early bolting genotypes with higher meristems were more susceptible to freezing damage, while late-bolting genotypes exhibited superior cold stress tolerance [15]. Lu conducted QTL mapping for bolting traits, clarifying their genetic basis [16]. Pang et al. demonstrated that foliar application of paclobutrazol reduced pre-winter bolting height and rate, thereby improving overwintering survival—indirectly supporting the link between bolting traits and cold tolerance [17]. Additionally, Minadakis et al. reported overlapping genetic networks between bolting-related traits and cold response pathways in *Brachypodium distachyon* [18]. Hu et al. showed that cumulative low-temperature exposure regulated stem internode elongation through vernalization, directly influencing bolting height and suggesting coordinated regulation of bolting development and cold response [19].

Although previous studies have explored bolting-related traits and cold tolerance, systematic research is still limited. Specifically, few studies have directly linked bolting height to cold tolerance in winter *B. napus* adapted to the dry–cold regions in northern China. In this study, a panel of 95 winter *B. napus* accessions from northern China was used in both spring and autumn sowing trials to evaluate the genetic stability of bolting height and to elucidate its relationship with thermo-sensitivity, cold tolerance, growth period, and key agronomic traits, with the goal to provide a theoretical foundation and practical selection criteria for breeding cold-hardy winter *B. napus* cultivars adapted to the dry–cold regions in northern China.

## 2. Results

### 2.1. Genetic Stability Analysis of Bolting Height in Winter B. napus

#### 2.1.1. Broad Variation in Bolting Height in Winter *B. napus*

Bolting height at the early flowering stage was measured in 95 winter *B. napus* accessions under spring and autumn sowing (Table 1). In the spring-sown trial, the mean bolting height was 27.76 cm, ranging from 0.00 to 94.70 cm with a high CV (87.40%), indicating wide phenotypic dispersion among accessions. Under autumn sowing, the mean bolting height increased to 64.87 cm (more than double the spring value), with a range of 10.78–107.50 cm and a much lower CV (23.48%). These results show that autumn sowing significantly raised the average bolting height and substantially reduced inter-accession variability.

**Figure 1:**
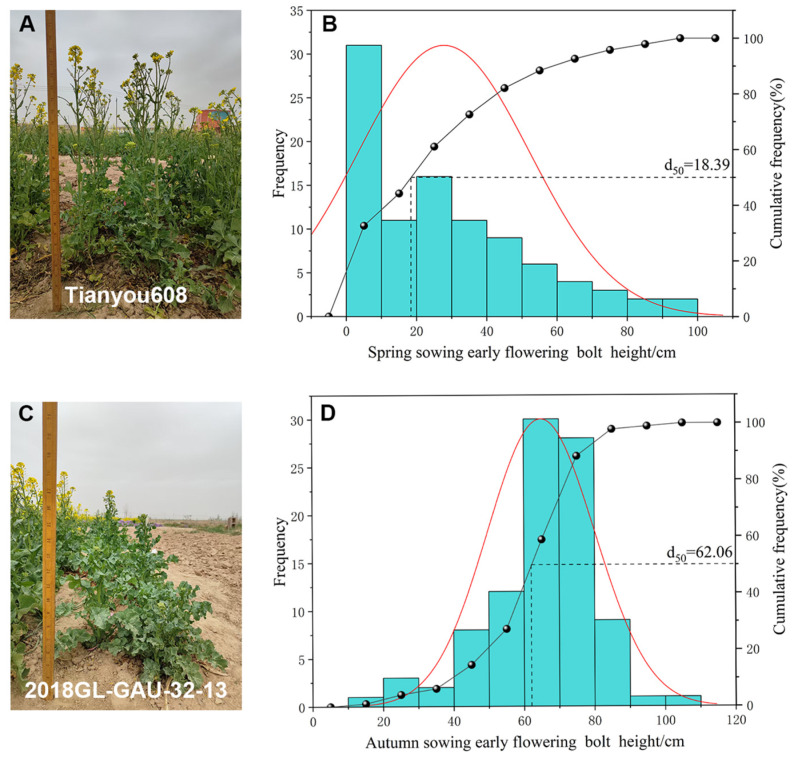
Frequency distribution of plant height in spring and autumn sowing at the initial flowering stage. Field phenotype of the high-stalk line Tianyou 608 (**A**) and the low-stalk line 2018GL-GAU-32-13 (**C**). Frequency distribution histogram of stalk height at the initial flowering stage in spring (**B**) and autumn (**D**) sowing.

Frequency distributions of bolting height under the two regimes are shown in Figure 1. Under spring sowing (Figure 1B), the distribution was broad and dispersed, with 50% of accessions ≤ 18.39 cm and no clear concentration peak, consistent with the high CV and confirming large phenotypic variation. In contrast, the autumn-sown distribution (Figure 1C) followed a normal curve: 50% of accessions were ≤62.06 cm, data concentrated in the 40–80 cm interval, and a clear unimodal pattern emerged. The distribution was narrower and more clustered than in spring, aligning with the lower CV.

#### 2.1.2. Stability of Bolting Height Across Sowing Trials

**Figure 2:**
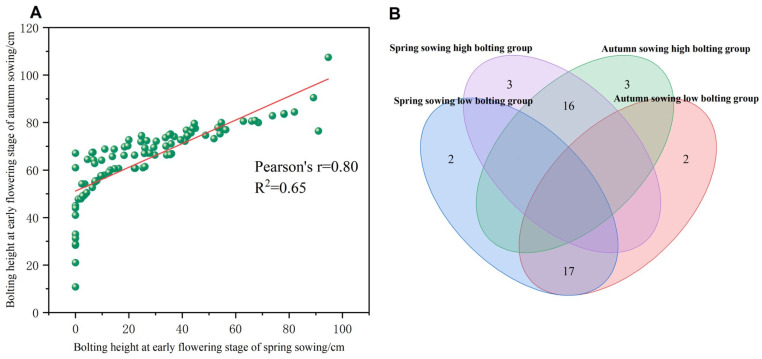
Consistency analysis of the bolting height ranking at the initial flowering stage between spring-sown and autumn-sown experiments. (**A**) Scatter plot of the correlation of plant height at the initial flowering stage between spring sowing and autumn sowing. (**B**) Venn diagram for the overlapping analysis of high and low plant height type varieties in spring and autumn sowing experiments.

To assess the consistency of bolting height at the early flowering stage between spring and autumn sowing, we analyzed the phenotypic stability across 95 winter *B. napus* accessions (Figure 2). A scatter plot of spring-sown versus autumn-sown bolting heights (Figure 2A) showed that most points lay above the diagonal line, indicating generally greater bolting height under autumn conditions. The two datasets were strongly positively correlated (*r* = 0.80), reflecting high ranking consistency across sowing trials.

To further confirm phenotype stability, we selected the 19 accessions with the lowest bolting height (“low-bolting type”) and the 19 with the highest bolting height (“high-bolting type”) from each sowing trial. A Venn diagram (Figure 2B) revealed substantial overlapping: 89.5% (17/19) spring low-bolting accessions were also classified as low-bolting in autumn, and 84.2% (16/19) spring high-bolting accessions remained high-bolting in autumn, indicating that accessions with either low or high bolting height in spring largely retained their phenotypic classification in autumn. Therefore, bolting height is a stable trait influenced little by sowing regime.

### 2.2. Correlation Analysis Between Bolting Height and Thermo-Sensitivity

#### 2.2.1. Thermo-Sensitivity Differences Among Accessions Based on Vernalization Index

Vernalization refers to the requirement of sustained exposure to low temperatures for normal bolting and flowering [20]. After spring sowing, clear differences in development were observed both among and within accessions, with plants classified into five distinct states: unbolted, bolted without buds, bolted with buds, flowering, and mature.

The vernalization index (VI), calculated as the proportion of plants that completed vernalization, reflects the low-temperature requirement of the population and directly indicates the level of thermo-sensitivity in winter *B. napus.* Based on VI values under spring sowing, accessions were grouped into five thermo-sensitivity grades (Table 2): Grade I (11 accessions, mean VI = 0.87), Grade II (12 accessions, mean VI = 0.65), Grade III (11 accessions, mean VI = 0.45), Grade IV (23 accessions, mean VI = 0.29), and Grade V (38 accessions, mean VI = 0.04). This grading confirms substantial variation in thermo-sensitivity across the 95 accessions, with VI values covering the full range from 0 to 1.0.

#### 2.2.2. Relationship Between Bolting Height and Thermo-Sensitivity

**Figure 3:**
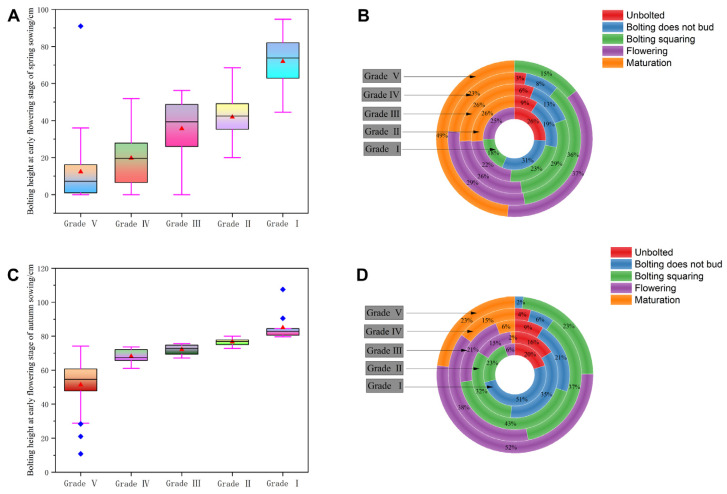
Differences in bolting height and developmental status based on different temperature sensitivities. Differences in bolting height (**A**) and developmental status (**B**) of materials with different thermo-sensitivities in the spring sowing experiment. Differences in bolting height (**C**) and developmental status (**D**) of materials with different thermo-sensitivities in the autumn sowing experiment.

In the spring-sowing trial, we assessed genotypic variation in bolting height at the early flowering stage and developmental progression among *B. napus* accessions (Figure 3A,B). Bolting height increased with greater vernalization responsiveness (higher vernalization index), and clear phenotypic stratification was observed across sensitivity grades (Figure 3A). A consistent developmental trend was observed, i.e., as thermo-sensitivity decreased, the proportions of non-bolting and bolting-but-non-budding plants declined, while the frequency of mature plants rose (Figure 3B). For example, in Grade I accessions (highest VI, lowest thermo-sensitivity), 26% remained non-bolted, 31% bolted without budding, and none reached maturity. In contrast, Grade V accessions (lowest VI, highest thermo-sensitivity) had no non-bolting or bolting-but-non-budding plants, with mature plants accounting for 49% of the population (Figure 3B).

We further evaluated this relationship in the autumn-sowing trial by measuring bolting height and developmental stage frequencies across the same thermo-sensitivity grades (Figure 3C,D). Similar to the spring trial, bolting height increased as thermo-sensitivity decreased (Figure 3C). However, the distribution of developmental stages differed between sowing trials. Under autumn sowing, lower thermo-sensitivity corresponded to fewer non-bolting and bolting-but-non-budding plants and more flowering and mature plants. In Grade I, 20% were non-bolting, 51% bolted without budding, 6% reached flowering, and none matured. In Grade V, non-bolting and bolting-but-non-budding plants fell to 0% and 2%, respectively, while flowering and mature plants increased to 52% and 23%, respectively (Figure 3D).

Together, these results revealed a clear relationship between bolting height and thermo-sensitivity: higher thermo-sensitivity is associated with reduced bolting growth and delayed reproductive development. Therefore, this trait could likely help winter *B. napus* avoid low-temperature stress, thereby improving cold stress tolerance.

### 2.3. Correlation Between Bolting Height and Cold Stress Tolerance

#### 2.3.1. Comprehensive Evaluation of Cold Tolerance

To comprehensively evaluate cold tolerance across 95 winter B. napus accessions, we normalized two key metrics—overwintering rate and LT_50_—and calculated a comprehensive cold tolerance score, which was then ranked (Table 3). Scores differed significantly among accessions, ranging from 0.00 to 2.00. Accession B62 exhibited the highest score (2.00), with an overwintering rate of 76% and an LT_50_ of −13.76 °C, indicating the strongest cold stress tolerance. In contrast, accession B95 had the lowest score (0.00), with an overwintering rate of 29.4% and an LT_50_ of −4.13 °C, reflecting the weakest cold stress tolerance.

#### 2.3.2. Correlation Between Bolting Height and Cold Tolerance

**Figure 4:**
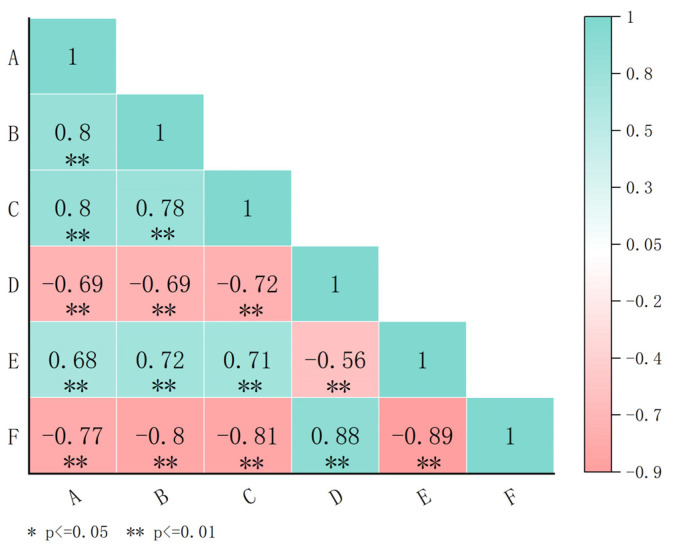
Heat map of correlation analysis between bolting height and cold stress tolerance. A and B indicate the bolting height at the initial flowering stage in spring and autumn sowing, respectively. C, D, E, and F indicate the vernalization index, overwintering rate, semi-lethal temperature, and comprehensive score of cold resistance, respectively. * and ** indicate a significant correlation at the 0.05 and 0.01 level, respectively. Green and red colors represent positive and negative correlations, respectively.

Correlation heatmap analysis revealed consistent negative relationships between early flowering bolting height and cold tolerance across sowing trials (Figure 4). Under both spring and autumn sowing, bolting height showed strong negative correlations with overwintering rate (*r* = −0.69 for both) and the composite cold tolerance score (spring: *r* = −0.77; autumn: *r* = −0.80) and a positive correlation with LT_50_ (spring: *r* = 0.68; autumn: *r* = 0.72). These results confirm that lower bolting height reliably predicts superior cold tolerance, regardless of sowing time. Notably, the VI was positively correlated with bolting height (*r* = 0.80 in spring, 0.78 in autumn) and LT_50_ (*r* = 0.71) and negatively correlated with overwintering rate (*r* = −0.72) and composite cold tolerance score (*r* = −0.81), further linking these traits.

### 2.4. Regulatory Mechanism of Bolting Height on Cold Resistance

#### 2.4.1. Differences in Shoot Tip Cell Microstructure Among Materials with Different Cold Resistances

**Figure 5:**
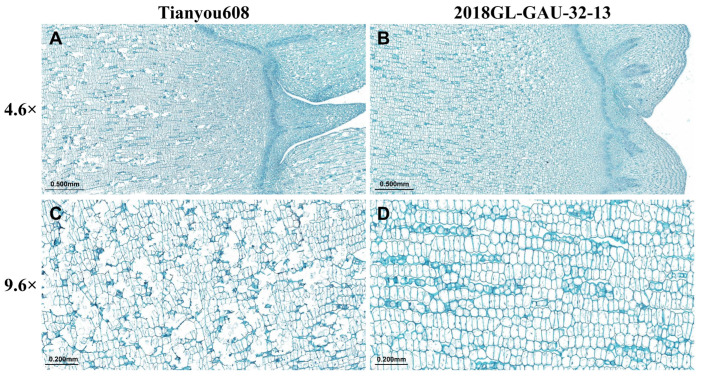
Paraffin sections of shoot apical meristem (longitudinal sections). Microscopic observation of Tianyou 608 (**A**) and 2018GL-GAU-32-13 (**B**) (4.6×), scale bar = 0.500 mm. Microscopic observation of Tianyou 608 (**C**) and 2018GL-GAU-32-13 (**D**) (9.6×), scale bar = 0.200 mm.

Tianyou 608 (high-bolting, low cold resistance) and 2018GL-GAU-32-13 (low-bolting, high cold resistance) were selected to investigate the shoot tip cell microstructure among materials with different cold resistances. Shoot apical meristems were sampled before overwintering and subjected to paraffin sectioning. Microscopic observations (Figure 5) showed that the shoot apex of Tianyou 608 was tall and sharp, indicating that it had entered the early reproductive growth stage (Figure 5A). The cells were large in volume, highly vacuolated, with thin cell walls and a loose arrangement, resulting in a fragile physical cold protection barrier of tissues (Figure 5C). By contrast, 2018GL-GAU-32-13 remained at the vegetative growth stage, presenting a flat and broad shoot apex (Figure 5B). Its cells were small and compact, with a high nucleoplasmic ratio and no obvious vacuoles, maintaining a stress-resistant meristem-like morphology (Figure 5D).

#### 2.4.2. Expression Patterns of Key Genes

**Figure 6:**
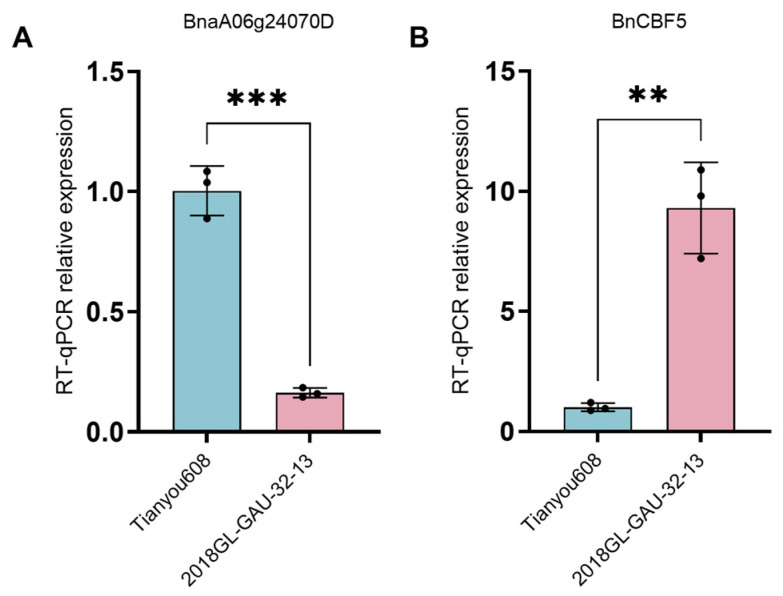
Relative expression levels of *BnaA06g24070D* (**A**) and *BnCBF5* (**B**) in high-stalk Tianyou 608 and low-stalk 2018GL-GAU-32-13. ** and *** indicate *p* < 0.01, and *p* < 0.001, respectively. All experiments were performed with three biological replicates, and similar results were obtained.

RT-qPCR was employed to detect the expression of the gibberellin biosynthesis gene *BnaA06g24070D* and the key cold resistance gene *BnCBF5* in the shoot apical meristems of Tianyou 608 and 2018GL-GAU-32-13. The results showed that the gibberellin biosynthesis gene *BnaA06g24070D* was significantly upregulated in Tianyou 608 (Figure 6A), whereas the expression of the key cold resistance gene *BnCBF5* was suppressed (Figure 6B). These results revealed an antagonistic relationship between growth and defense at the transcriptional level.

#### 2.4.3. Regulatory Effects of Exogenous Hormones on the Relationship Between Bolting Height and Cold Tolerance

**Figure 7:**
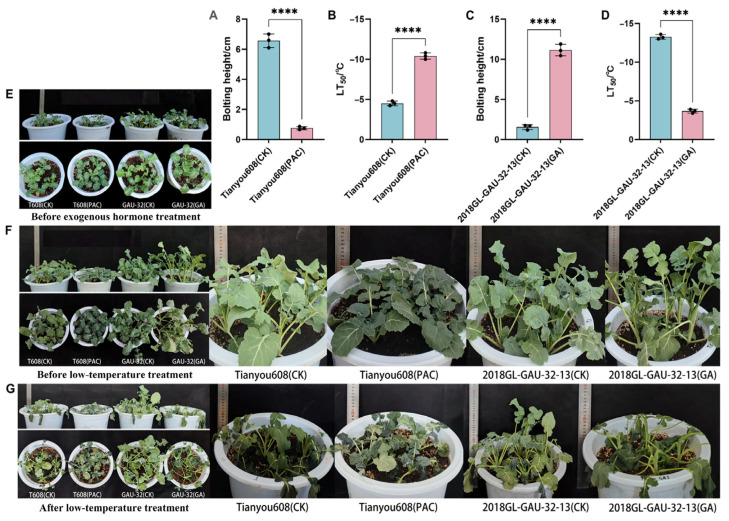
Changes in stalk height and cold resistance after exogenous hormone treatment. Changes in the bolting height (**A**) and cold resistance (**B**) of Tianyou 608 after 15 days of paclobutrazol treatment. Changes in the bolting height (**C**) and cold resistance (**D**) of 2018GL-GAU-32-13 after 15 days of gibberellin treatment. Phenotypic photographs of the four groups before exogenous hormone treatment (**E**), before low-temperature treatment (**F**), and after low-temperature treatment (**G**). **** indicates a significant difference at the *p* < 0.0001 level. All experiments were performed in three biological replicates with similar results.

To verify that low bolting height is the key determinant of improved cold tolerance, exogenous hormones were applied to the selected extreme accessions. After 15 days of treatment, bolting height was measured in the control group using distilled water and treatment groups using exogenous hormone. Meanwhile, relative electrical conductivity (REC) under different temperature treatments was determined, and the semi-lethal temperature was calculated. The results showed that paclobutrazol (PAC) treatment significantly reduced the bolting height of the high-bolting accession Tianyou 608 (Figure 7A). Moreover, the semi-lethal temperature of the PAC treatment group was markedly lower than that of the control group (Figure 7B). PAC application alleviated cell membrane damage under cold stress in the originally cold-sensitive accession, thus enhancing cold tolerance. Conversely, exogenous gibberellin (GA) application induced elongation of the low-bolting accession 2018GL-GAU-32-13 (Figure 7C), accompanied by a significant increase in semi-lethal temperature (Figure 7D) and a decline in cold tolerance.

In summary, the low-bolting trait improves cold tolerance by maintaining compact stress-resistant shoot apical structures under vegetative growth and relieving the inhibition of the key cold resistance gene *BnCBF5* via downregulated expression of gibberellin biosynthesis genes. Exogenous hormone regulation experiments further confirm that bolting height directly determines plant overwintering cold tolerance, and low bolting height is the critical determinant for enhancing cold tolerance in winter *B. napus*.

### 2.5. Correlation Between Bolting Height, Growth Period, and Agronomic Traits

#### 2.5.1. Correlation Between Bolting Height and Growth Period

The analysis of growth period between low-bolt type and high-bolt type showed that the key growth stages were prolonged in low-bolting accessions relative to high-bolting ones (Table 4): the bolting stage was prolonged by 3.05 days, the budding stage by 4.36 days, the early flowering stage by 3.57 days, and the total growth period by 5.67 days. Correlation analysis revealed highly significant negative correlations between bolting height and each of these stages, with the exception of the silique development–maturation stage: bolting stage (*r* = −0.73), budding stage (*r* = −0.75), early flowering stage (*r* = −0.86), and total growth period (*r* = −0.68). In contrast, the silique development–maturation stage showed no significant correlation with bolting height (*r* = −0.41). These results indicated a close relationship between bolting height and growth progression in winter *B. napus*, i.e., reduced bolting height is associated with significantly longer durations of bolting, budding, early flowering, and the total growth cycle. Among these stages, early flowering exhibited the strongest response to variation in bolting height.

#### 2.5.2. Analysis of Agronomic Traits

Analysis of 10 agronomic traits during the 2023–2024 growing season revealed statistically significant differences between low- and high-bolting winter *B. napus* accessions collected from the dry–cold regions of northern China (Table 5). High-bolting accessions showed higher values in nine traits: plant height, branch initiation height, number of primary branches, effective main inflorescence length, number of pods on the main inflorescence, total pods per plant, silique length, seeds per silique, and seed yield per plant. In contrast, low-bolting accessions had a significantly higher 1000-seed weight (4.29 g vs. 3.70 g). Inter-annual CVs were greater in high-bolting materials, particularly for 1000-seed weight (22.70%) and seed yield per plant (23.42%). Low-bolting accessions consistently exhibited lower CVs across all traits, reflecting greater phenotypic stability. These results indicate that low-bolting genotypes offer not only higher 1000-seed weight but also enhanced trait stability—key attributes for reliable yield performance in the variable dry–cold environments of northern China.

### 2.6. Screening of Elite Varieties

To screen elite winter *B. napus* varieties adapted to arid and cold regions in northern China, a comprehensive evaluation and selection were conducted on 95 test accessions in this study (Table 6). The top three accessions (B62, B74, and B1) performed excellently under both spring and fall planting conditions. Specifically, these three accessions showed complete non-bolting in spring sowing, indicating high vernalization and extremely strong winter habits. In fall sowing, their bolt height remained at an extremely low level, which effectively alleviated freezing damage during overwintering. In addition, they also exhibited superior performance in other key agronomic and stress-resistant traits, including higher 1000-seed weight, smaller annual fluctuation, an overwintering rate above 74%, and a semi-lethal temperature (LT_50_) lower than −12.98 °C, demonstrating outstanding cold tolerance and stress resistance. These three accessions can be used as important germplasm resources for breeding new winter *B. napus* varieties suitable for arid and cold regions in northern China and provide a valuable genetic basis for breeding cold-resistant and high-yield winter rapeseed in this region.

## 3. Discussion

### 3.1. Cytological and Physiological Basis of Growth–Defense Trade-Off

This study revealed a significant negative correlation between bolting height and cold tolerance in winter *B. napus*, providing direct evidence for the plant growth–defense trade-off theory at the cytological and hormonal regulation levels. Under the low-temperature stress in northern winters, the key to a plant’s successful overwintering lies in its ability to actively terminate its growth programs and establish corresponding physical and physiological cold tolerance mechanisms before the onset of low temperatures. The cold susceptibility of high-bolting accessions precisely originates from the failure of this transition mechanism. From a cytological perspective, the differences in cold tolerance are primarily reflected in the morphological characteristics of the shoot apical meristems. The microscopic observation results presented in this study showed that the shoot tip cells of cold-tolerant low-bolting accessions are small in size, compact in structure, have a high nucleoplasmic ratio, and are tightly arranged. In contrast, cold-sensitive high-bolting accessions exhibited a larger cell volume, higher vacuolation, thinner cell walls, and a looser cellular arrangement. Such morphological differences directly determine the physical potential of tissues to resist freezing injury. Previous studies have confirmed that extracellular ice crystals are major causes of mechanical freezing damage. Water expands by approximately 9% in volume when it freezes, causing severe compression and dehydration stress to surrounding cells. Pearce et al. [21] reported that small cellular tissues could effectively narrow the intercellular space, thereby reducing the formation and expansion of ice crystals. Meanwhile, small cells possess a higher cell wall surface area to volume ratio, endowing the tissues with greater mechanical strength to withstand the cell shrinkage and tension induced by extracellular freezing and avoid cell membrane damage. This view is further supported by Rajashekar and Burke [22], who demonstrated that dense tissues with rigid cell walls generated stronger cellular tension during extracellular freezing, which inhibited excessive cellular dehydration and enhanced the tissue’s tolerance to freezing. Therefore, low-bolting accessions resist frost damage by maintaining small and compact cellular structures. At the hormonal regulation level, gibberellin (GA)-mediated growth inhibition is central to cold acclimation and the establishment of cold tolerance. The present study revealed that cold acclimation essentially refers to the suppression of GA biosynthesis in plants, thereby halting stem elongation and reallocating resources from growth to defense. Gene expression analysis verified that the GA biosynthesis gene *BnaA06g24070D* was highly expressed in high-bolting accessions, whereas the key cold-tolerance gene *BnCBF5* was significantly suppressed. These results are highly consistent with previous studies on winter wheat. Vankova et al. [23] pointed out that cold-tolerant winter wheat lines significantly reduced the content of endogenous active GA during cold acclimation, thereby inhibiting elongation growth, whereas the recovery of GA levels is closely associated with deacclimatization. Further experiments with exogenous hormone treatment validated this causal relationship. The application of paclobutrazol (PAC), a GA biosynthesis inhibitor, effectively reduced bolting height and significantly improved cold tolerance in high-bolting accessions. Conversely, exogenous application of GA induced elongation in low-bolting accessions and impaired their cold tolerance. These findings suggest that the poor cold tolerance of high-bolting accessions arises from their insensitivity to low-temperature signals and their inability to effectively downregulate the GA biosynthesis pathway. Sustained GA signaling not only promotes stem elongation but also inhibits the activation of the CBF cold-responsive pathway via transcriptional antagonism, thereby preventing plants from establishing an effective cold defense system [24]. In summary, the cold tolerance in winter rapeseed is essentially a consequence of the trade-off between growth and defense. Low-bolting accessions actively suppress GA biosynthesis and maintain a small, compact cellular wall structure, thereby achieving a shift in physiological strategy from growth-dominant to defense-dominant status. Conversely, in high-bolting accessions, GA signaling disorders lead to continuous plant growth and a reduction in overwintering capacity. The elucidation of this mechanism provides clear physiological indicators and molecular targets for breeding cold-tolerant winter rapeseed in northern China.

### 3.2. Breeding Application of Low Bolting Height in Winter B. napus

The arid and cold regions in northern China are potential core areas for the northward expansion of China’s winter rapeseed industry. However, the low overwintering survival rate has long limited the promotion and application of winter *B. napus* in the region. Breeding new varieties that are cold-tolerant with stable yields has become the key to solving this industrial bottleneck [6,25,26]. The results showed that bolting height is a stable trait less affected by the environment. In spring and autumn sowing trials, the ranking of bolting height among varieties was highly consistent. In addition, 89.47% of spring-sown low-bolting accessions stably maintained the low-bolting phenotype in the autumn-sown trial. This characteristic indicates that precise selection based on bolting height can be carried out in early breeding populations without complicated environmental identification, thereby greatly reducing the dependence of cold-tolerance breeding on the environment and effectively shortening the breeding cycle. The present study revealed a highly significant negative correlation between bolting height and cold tolerance: the lower the bolting height, the stronger the cold tolerance. Although previous studies have observed that the growing point height of cold-tolerant winter *B. napus* varieties is significantly lower than that of cold-sensitive varieties, the underlying cold tolerance mechanisms are completely different. Growing point height determines whether the meristem can be buried in soil, thus avoiding low temperatures via soil buffering and preventing the exposed meristem from being damaged by frost. In contrast, bolting height affects the elongated, tender floral stem tissues that are fully exposed to the chilly air. These tissues have a high degree of cell differentiation and are far less hardy than vegetative tissues. A higher bolting height means a larger area of exposed tender stem, making the entire stem more susceptible to frost damage under extreme low temperatures in arid and cold northern regions [27]. More importantly, the present study overcame the limitations of correlation analysis and elucidated the regulatory mechanism at cytological and molecular levels. Low-bolting accessions maintain the structure of the shoot apical meristem under vegetative growth, characterized by small and compact cells with a high nucleoplasmic ratio, thus forming a robust physical barrier against cold. Meanwhile, the low expression of the GA biosynthesis gene *BnaA06g24070D* in low-bolting accessions relieves its transcriptional inhibition of the key cold-tolerance gene *BnCBF5*, achieving synergistic regulation of growth and defense [24,28]. Through reverse regulation experiments with exogenous paclobutrazol and gibberellin, the causal regulatory effect of bolting height on cold tolerance in winter *B. napus* was verified for the first time. This confirmed that low bolting height is not an associated trait of cold tolerance, but rather a direct regulatory factor. In terms of agronomic adaptability, the low-bolting trait exhibits unique stable-yield advantages for cold-region breeding. Although the yield per plant of low-bolting accessions is slightly lower than that of high-bolting accessions, the interannual stability of their agronomic traits is significantly superior. The coefficient of variation for the yield per plant of low-bolting accessions is only half that of high-bolting accessions. Furthermore, low-bolting accessions also exhibit a significant advantage in 1000-seed weight, averaging 4.29 g, which is significantly higher than the 3.70 g of high-bolting accessions. In agricultural production in arid and cold northern regions, where high-bolting accessions have higher yield potential, their weak cold tolerance leads to a low overwintering survival rate, dramatic yield fluctuations, and difficulty in achieving stable seed yield. In contrast, low-bolting accessions can ensure overwintering survival and maintain stable yields in low-temperature environments, which is of more practical value for agricultural production in arid and cold northern regions than simply pursuing high yields [29]. Meanwhile, the moderately prolonged growth period of low-bolting accessions allows them to better adapt to the light and temperature resources in northern China, enabling them to fully utilize the light and temperature conditions in spring for grain filling and thus compensate for yield losses caused by the extended growing season. Therefore, low bolting is a safe and feasible selection indicator for the improvement of winter *B. napus* in arid and cold northern regions in China.

## 4. Materials and Methods

### 4.1. Test Materials

A total of 95 winter-type *B. napus* accessions, comprising cultivars and inbred lines, were used in this study. The germplasm was developed by Gansu Agricultural University and the Tianshui Academy of Agricultural Sciences (Table S1).

### 4.2. Experimental Site and Design

Field trials were conducted during the 2022–2023 growing season at the Rapeseed Engineering Center Experimental Station, located in Yongdeng County, Gansu Province, China (36°48′ N, 103°25′ E; elevation 1960 m). The site has a cold–arid climate typical of northern China, with an annual average temperature of 5.9 °C, an average temperature during January of −8.6 °C, and annual precipitation of 280 mm.

Two distinct sowing trials were implemented: autumn sowing and spring sowing. Plot dimensions and agronomic management were consistent across both regimes. Each plot consisted of three rows, each 1 m in length, with 0.3 m between rows and 0.15 m between plants. Standard local practices for fertilization, irrigation, and pest control were uniformly applied.

For the spring sowing experiment, manual sowing was performed on 30 March 2023. Seedlings were thinned, weeded, and established following germination. All cultivars were harvested at full physiological maturity on 25 September 2023.

For the autumn sowing experiment, the sowing dates were 31 August 2022 and 17 August 2023. To enhance winter survival, a single irrigation event occurred in late November prior to soil freezing. Plants resumed growth in mid-March of the following year, received supplemental irrigation and topdressing in mid-April, and were harvested at full physiological maturity.

### 4.3. Phenotypic Trait Evaluation

#### 4.3.1. Bolting Height

Bolting height was measured at the initial flowering stage, defined as the time when approximately 25% of plants in the experimental field had initiated flowering. Measurements were taken on 12 August 2023 for the spring-sown trial and on 10 April 2023 for the autumn-sown trial. For each plot, the vertical distance from the soil surface at the plant base to the apex of the elongating inflorescence was recorded for 10 representative plants using a standard ruler. To ensure the representativeness of the selected plants, individuals with uniform vigor were screened by the following criteria: (1) consistent plant height (within ±5% of the average height of the whole plot), (2) intact and disease-free aboveground tissues, (3) the synchronous inflorescence elongation stage, and (4) no obvious signs of abiotic stress (e.g., drought, nutrient deficiency). The mean value per plot was used as the bolting height for the corresponding accession.

#### 4.3.2. Cold Stress Tolerance Assessment

Cold tolerance was evaluated using two complementary approaches: field-based overwintering rate and laboratory-determined semi-lethal temperature (LT_50_).

Overwintering rate: For the autumn-sown trial in 2022, the plant number per plot was counted immediately before winter (late November 2022) and after spring regreening (mid-March 2023). The overwintering rate was calculated as follows:

(1) $$Overwintering\;rate\left(\%\right)\;=\;\frac{N_{after\;regreening}}{N_{before\;winter}}\;\times 100\%$$

Semi-lethal temperature (LT_50_): The electrolyte leakage method was employed following a modified protocol [30]. In early September 2022, plants were cultivated in pots under natural conditions. At the seven-leaf stage, the third fully expanded leaf was excised from each plant between 09:00 and 10:00 to minimize diurnal variation. Leaf discs (approximately 8 mm in diameter) were collected, avoiding major veins, and six discs were placed in a 10 mL tube containing deionized water. Samples were exposed to five temperature treatments (3 °C, 0 °C, −3 °C, −6 °C, and −9 °C) for 3 h in a programmable low-temperature circulator, with three replicates per temperature. Following treatment, tubes were shaken for 3 h at room temperature, and the initial electrical conductivity (*R*_1_) was measured using a DJS-1D conductivity meter (Leici, Shanghai, China). Tubes were then autoclaved at 120 °C for 40 min to release all electrolytes, shaken again for 3 h, and the final conductivity (*R*_2_) was measured. Relative electrolyte conductivity (REC) was calculated as follows:

$$REC\left(\%\right)\;=\;\frac{R_{1}}{R_{2}}\;\times 100\%$$

REC values were fitted to a logistic regression model:

(2) $$y\;=\;\frac{k}{\left(1\;+\;{ae}^{bt}\right)}$$

where *y* represents REC, *t* is the treatment temperature, and *k*, *a*, and *b* are model parameters. The equation was linearized as follows:

(3) $$\ln\left(\frac{k\;-\;y}{y}\right)=lna\;-\;bt$$

(4) $$y^{\prime }=ln(\frac{k-y}{y})$$

Parameters were estimated via linear regression. LT_50_ was then derived as follows:

(5) $${LT}_{50}\;=\;\frac{lna}{b}$$

Comprehensive composite cold tolerance score: To integrate both field and laboratory metrics, a normalized composite cold tolerance score was generated for each accession. Overwintering rate was positively normalized, and LT_50_ was negatively normalized using the following formulas:

(6) $$U_{ij}\;=\;\frac{X_{ij}\;-\;X_{jmin}}{X_{jmax}\;-\;X_{jmin}}\;(\mathrm{Positive normalization})$$

(7) $$U_{ij}=\frac{X_{jmax}-X_{ij}}{X_{jmax}-X_{jmin}}\;(\mathrm{Negative normalization})$$

where *U*_ij_ is the normalized value for accession i and trait j, *X*_ij_ is the measured value, and *X*_jmax_ and *X*_jmin_ are the maximum and minimum values for trait j, respectively. The final composite cold tolerance score was computed as follows:

Score = *U*_overwintering_ + *U*_LT50_ (8)

Higher scores indicate stronger cold stress tolerance.

#### 4.3.3. Thermo-Sensitivity Evaluation

Thermo-sensitivity, reflecting the vernalization requirement, was assessed at initial flowering. Plants were classified into five developmental grades: Grade 1, no bolting; Grade 2, bolted but without visible buds; Grade 3, bolted with buds; Grade 4, flowering; and Grade 5, mature. A vernalization index (VI) was calculated for each cultivar using a modified disease index formula [31]:

(9) $$VI=\;\frac{\sum (Number\;of\;plants\;at\;each\;grade\;\times \;Corresponding\;grade\;value)}{\left(Total\;number\;of\;surveyed\;plants\;\times \;5\right)}$$

Based on VI values, cultivars were assigned to one of five thermo-sensitivity grades: Grade I, 0.8 ≤ VI ≤ 1.0; Grade II, 0.6 ≤ VI < 0.8; Grade III, 0.4 ≤ VI < 0.6; Grade IV, 0.2 ≤ VI < 0.4; and Grade V, 0 ≤ VI < 0.2.

#### 4.3.4. Observation of Paraffin Sections

The shoot apical meristems of Tianyou 608 and 2018GL-GAU-32-13 were sampled for paraffin section preparation, which was carried out using the method described by Fan et al. [32]. Sections were observed and photographed using a Nikon Eclipse 80i microscope (Nikon, Tokyo, Japan).

#### 4.3.5. Extraction of RNA and Synthesis of cDNA

Total RNA was extracted from the shoot apical meristems, hypocotyls, roots, and leaves using a rapid plant tissue RNA extraction kit (Tiangen, Beijing, China). RNA integrity and purity were detected by 1% agarose gel electrophoresis. Reverse transcription was performed when the RNA concentration and quality met the requirements. First-strand cDNA was synthesized using FastKing one-step removal of genomic DNA first-strand cDNA synthesis master mix. A 20 μL reaction system was established with 50 ng to 2 μg of total RNA, 4 μL 5× FastKing-RT SuperMix, and RNase-Free ddH_2_O. The reverse transcription reaction procedure was set as follows: 42 °C for 15 min, 95 °C for 3 min.

#### 4.3.6. RT-qPCR Analysis

##### Primer Design

The CDS region of the gene was obtained using the Sequence Fetch tool on the BnIR database (https://yanglab.hzau.edu.cn/, accessed on 1 February 2026), with the ZS11.v0 reference genome as the internal control. Gene-specific primers were designed and synthesized by Sangon Biotech (Shanghai, China), and the primer sequences are listed in Table 7.

##### RT-qPCR Assay

RT-qPCR assays were performed in accordance with the instructions of the FastReal SYBR Green qPCR Master Mix kit (TIANGEN Biotech, Beijing, China). The PCR thermal cycling program was set as follows: pre-denaturation at 95 °C for 2 min; denaturation at 95 °C for 5 s; and annealing at 60 °C for 15 s for 40 cycles. Three technical replicates were set for each sample. The relative expression levels of genes were calculated using the 2^−ΔΔCt^ method [33,34].

#### 4.3.7. Exogenous Hormone Treatment

Exogenous hormone treatment was applied to five-leaf-stage seedlings. A total of 150 mg/L paclobutrazol (PAC, an elongation inhibitor) was sprayed on the high-bolt-height material Tianyou 608, while 200 mg/L gibberellin A (GA, an elongation promoter) was sprayed on the low-bolt-height material 2018GL-GAU-32-13. A few drops of dish soap were added as a surfactant to help the solution adhere to the leaves. After 15 days of treatment, low-temperature stress treatment was performed, and the relative electrical conductivity was measured.

#### 4.3.8. Growth Period Traits

Key phenological stages were recorded for each cultivar: sowing date, bolting date, budding date, first-flower date, and maturity date (defined as the time when ≥75% of siliques had ripened). From these records, the following intervals were derived: days from sowing to bolting, days from sowing to budding, days from sowing to flowering, days from flowering to maturity, and total growth period (sowing to maturity). Mean values across replicates were used for subsequent analysis.

#### 4.3.9. Agronomic Trait Measurements

At maturity, six plants in the middle row of each plot were individually harvested. The following traits were measured: plant height (cm), branch initiation height (cm), number of primary branches, length of main inflorescence (cm), pod number on main inflorescence, total pods per plant, pod length (cm), seeds per pod, 1000-seed weight (g), and seed yield per plant (g). To assess trait stability across seasons, the coefficient of variation (CV) was calculated for each trait as follows:

(10) $$CV\left(\%\right)\;=\;\frac{SD}{Mean}\;\times \;100\%$$

### 4.4. Statistical Analysis

All phenotypic data were organized and preliminarily processed using Microsoft Excel 2021. Statistical analyses, including Pearson’s correlation analysis and one-way analysis of variance (ANOVA), were conducted using IBM SPSS Statistics 26. Graphical representations of the data were prepared using Origin 2024 (OriginLab Corporation, Northampton, MA, USA).

## 5. Conclusions

This study systematically explored the correlations between bolting height and temperature sensitivity, cold tolerance, growth period, and other agronomic traits through both spring and autumn sowing trials. The results showed that bolt height is a relatively stable trait, exhibiting a consistent variation trend in spring and autumn sowing trials. Vernalization index measurements indicated a significant correlation between bolting height and temperature sensitivity: the stronger the temperature sensitivity, the lower the bolt height. Further research revealed a close correlation between bolt height and cold tolerance: varieties with lower bolt height exhibited stronger cold tolerance. This study elucidated the regulatory mechanisms of low bolting height and cold tolerance at cellular and molecular levels and preliminarily verified the causal regulatory role of bolting height on cold tolerance through exogenous hormone treatment. Low bolting height was found to not only enhance cold tolerance but also be closely related to growth duration and agronomic traits. Bolting height was negatively correlated with each growth stage. The stability of agronomic traits in low-bolting accessions was markedly superior to that of high-bolting accessions, demonstrating stronger environmental adaptability. Therefore, low bolting height is an important target trait and selection indicator for improving the cold tolerance of winter *B. napus* in northern China.

## Figures and Tables

**Table 1:** Analysis of variation in bolting height at the early flowering stage in the spring sowing test and the autumn sowing test.

Experiment	Character	Mean ± SD	Min	Max	CV/%
Spring sowing test	Bolting height at early flowering stage/cm	27.76 ± 24.38	0.00	94.70	87.40
Autumn sowing test	Bolting height at early flowering stage/cm	64.87 ± 15.23	10.78	107.50	23.48

**Table 2:** Thermo-sensitivity classification of test materials based on vernalization index.

Thermo-Sensitivity Grade	Grading Standard	Mean Value of Vernalization Index	Number of Test Materials	Breed Number
Grade I	0.8 ≤ VI ≤ 1	0.87	11	B5, B8, B45, B46, B75, B76, B83, B84, B90, B93, B95
Grade II	0.6 ≤ VI < 0.8	0.65	12	B3, B4, B6, B39, B56, B60, B79, B80, B82, B88, B91, B92
Grade III	0.4 ≤ VI < 0.6	0.45	11	B19, B40, B55, B58, B59, B61, B71, B72, B85, B86, B94
Grade IV	0.2 ≤ VI < 0.4	0.29	23	B9, B11, B21, B23, B29, B30, B32, B37, B38, B44, B47, B49, B50, B51, B54, B57, B64, B65, B77, B78, B81, B87, B89
Grade V	0 ≤ VI < 0.2	0.04	38	B1, B2, B7, B10, B12, B13, B14, B15, B16, B17, B18, B20, B22, B24, B25, B26, B27, B28, B31, B33, B34, B35, B36, B41, B42, B43, B48, B52, B53, B62, B63, B66, B67, B68, B69, B70, B73, B74

Note: The “B” prefix indicates the unified identifier for the batch of winter-type *B. napus* accessions used in this study, which has been consistently used in our breeding program and field trial records to ensure data traceability.

**Table 3:** The comprehensive score and ranking of cold stress tolerance of the 95 test materials.

			Normalized							Normalized			
No.	Overwintering Rate/%	LT_50_/°C	Overwintering Rate	LT_50_	Comprehensive Score	Ranking	No.	Overwintering Rate/%	LT_50_/°C	Overwintering Rate	LT_50_	Comprehensive Score	Ranking
B62	76.00	−13.76	1.00	1.00	2.00	1	B87	53.00	−8.77	0.51	0.48	0.99	49
B74	75.00	−13.31	0.98	0.95	1.93	2	B77	53.00	−8.64	0.51	0.47	0.97	50
B1	74.00	−12.98	0.96	0.92	1.88	3	B35	66.80	−5.64	0.80	0.16	0.96	51
B20	72.00	−12.95	0.91	0.92	1.83	4	B23	52.00	−8.49	0.48	0.45	0.94	52
B43	70.50	−12.89	0.88	0.91	1.79	5	B9	57.40	−7.24	0.60	0.32	0.92	53
B28	70.00	−12.48	0.87	0.87	1.74	6	B60	51.00	−8.36	0.46	0.44	0.90	54
B69	70.40	−12.04	0.88	0.82	1.70	7	B53	66.50	−5.09	0.80	0.10	0.90	55
B52	70.60	−11.89	0.88	0.81	1.69	8	B26	54.00	−7.56	0.53	0.36	0.88	56
B16	68.00	−12.11	0.83	0.83	1.66	9	B51	56.20	−6.78	0.58	0.28	0.85	57
B24	66.00	−11.91	0.79	0.81	1.59	10	B94	48.70	−8.26	0.41	0.43	0.84	58
B34	66.00	−11.83	0.79	0.80	1.58	11	B42	52.00	−7.51	0.48	0.35	0.84	59
B41	70.00	−10.14	0.87	0.62	1.50	12	B78	48.60	−8.14	0.41	0.42	0.83	60
B36	64.80	−11.09	0.76	0.72	1.48	13	B49	65.30	−4.64	0.77	0.05	0.82	61
B33	70.00	−9.87	0.87	0.60	1.47	14	B47	48.00	−8.08	0.40	0.41	0.81	62
B2	60.40	−11.73	0.67	0.79	1.45	15	B63	51.30	−7.31	0.47	0.33	0.80	63
B12	69.80	−9.64	0.87	0.57	1.44	16	B59	47.20	−7.97	0.38	0.40	0.78	64
B18	64.00	−10.77	0.74	0.69	1.43	17	B7	47.00	−7.92	0.38	0.39	0.77	65
B15	60.00	−11.56	0.66	0.77	1.43	18	B50	52.80	−6.51	0.50	0.25	0.75	66
B13	59.00	−11.55	0.64	0.77	1.41	19	B22	48.70	−7.21	0.41	0.32	0.73	67
B14	58.40	−11.41	0.62	0.76	1.38	20	B65	62.00	−4.45	0.70	0.03	0.73	68
B89	62.00	−10.47	0.70	0.66	1.36	21	B86	45.60	−7.83	0.35	0.38	0.73	69
B66	68.20	−8.88	0.83	0.49	1.33	22	B80	42.80	−7.62	0.29	0.36	0.65	70
B19	60.40	−10.45	0.67	0.66	1.32	23	B72	42.50	−7.44	0.28	0.34	0.62	71
B31	56.60	−11.18	0.58	0.73	1.32	24	B91	42.00	−7.37	0.27	0.34	0.61	72
B67	45.00	−13.44	0.33	0.97	1.30	25	B79	42.00	−7.34	0.27	0.33	0.60	73
B70	47.00	−12.98	0.38	0.92	1.30	26	B71	41.60	−7.31	0.26	0.33	0.59	74
B57	59.20	−10.22	0.64	0.63	1.27	27	B85	41.60	−7.14	0.26	0.31	0.57	75
B17	56.50	−10.77	0.58	0.69	1.27	28	B3	41.40	−6.99	0.26	0.30	0.55	76
B64	58.30	−10.21	0.62	0.63	1.25	29	B82	41.00	−6.97	0.25	0.29	0.54	77
B37	58.00	−10.11	0.61	0.62	1.23	30	B92	40.80	−6.88	0.24	0.29	0.53	78
B73	42.00	−13.24	0.27	0.95	1.22	31	B88	38.00	−6.76	0.18	0.27	0.46	79
B11	57.40	−9.84	0.60	0.59	1.19	32	B54	45.60	−4.88	0.35	0.08	0.43	80
B58	57.30	−9.78	0.60	0.59	1.19	33	B39	38.00	−6.44	0.18	0.24	0.42	81
B38	57.00	−9.55	0.59	0.56	1.16	34	B56	37.60	−6.36	0.18	0.23	0.41	82
B61	57.00	−9.51	0.59	0.56	1.15	35	B4	37.20	−6.12	0.17	0.21	0.37	83
B48	56.40	−9.52	0.58	0.56	1.14	36	B90	37.00	−6.02	0.16	0.20	0.36	84
B81	56.60	−9.47	0.58	0.55	1.14	37	B6	36.60	−5.73	0.15	0.17	0.32	85
B10	39.00	−13.05	0.21	0.93	1.13	38	B93	36.00	−5.67	0.14	0.16	0.30	86
B27	42.00	−12.29	0.27	0.85	1.12	39	B84	36.00	−5.33	0.14	0.12	0.27	87
B32	68.00	−6.89	0.83	0.29	1.11	40	B45	34.60	−5.11	0.11	0.10	0.21	88
B68	41.20	−12.38	0.25	0.86	1.11	41	B46	34.20	−5.02	0.10	0.09	0.20	89
B21	56.20	−9.07	0.58	0.51	1.09	42	B5	34.00	−4.94	0.10	0.08	0.18	90
B55	56.00	−9.02	0.57	0.51	1.08	43	B76	33.00	−4.84	0.08	0.07	0.15	91
B25	38.00	−12.67	0.18	0.89	1.07	44	B83	33.00	−4.54	0.08	0.04	0.12	92
B29	59.00	−8.25	0.64	0.43	1.06	45	B8	32.40	−4.51	0.06	0.04	0.10	93
B44	55.00	−8.92	0.55	0.50	1.05	46	B75	32.10	−4.17	0.06	0.00	0.06	94
B40	55.00	−8.84	0.55	0.49	1.04	47	B95	29.40	−4.13	0.00	0.00	0.00	95
B30	54.20	−8.81	0.53	0.49	1.02	48							

**Table 4:** Analysis of growth period between the low-bolt type and the high-bolt type. ** indicate *p* < 0.01.

Character	Low-Bolt Type	High-Bolt Type	Correlation with Bolting Height
Bolting period	229.68 ± 2.13	226.63 ± 2.30	−0.73 **
Budding period	235.21 ± 3.38	230.85 ± 2.52	−0.75 **
Early flowering period	257.11 ± 3.04	253.54 ± 3.00	−0.86 **
The mature period of pod development	61.00 ± 2.19	59.18 ± 2.93	−0.41
Growth period	318.13 ± 4.47	312.46 ± 6.02	−0.68 **

**Table 5:** Performance of 10 agronomic traits across two growing seasons (2023 and 2024).

Character	Group	2023	2024	Mean ± SD	CV/%
Plant height/cm	Low-bolt type	118.50 ± 8.20	122.30 ± 7.80	120.40 ± 8.01	6.65
	High-bolt type	145.60 ± 12.50	148.80 ± 11.90	147.20 ± 12.25	8.32
Branch initiation height/cm	Low-bolt type	18.30 ± 2.00	20.20 ± 1.70	19.25 ± 1.88	9.77
	High-bolt type	35.80 ± 4.70	38.50 ± 4.30	37.15 ± 4.55	12.25
First branch number	Low-bolt type	9.33 ± 0.70	7.33 ± 0.60	8.55 ± 0.65	7.60
	High-bolt type	13.33 ± 1.40	12.00 ± 1.20	12.73 ± 1.27	9.98
Effective length of main inflorescence/cm	Low-bolt type	35.60 ± 4.20	37.20 ± 3.80	36.40 ± 4.03	11.07
	High-bolt type	42.80 ± 5.50	44.30 ± 5.10	43.55 ± 5.33	12.24
Effective silique number of main inflorescence	Low-bolt type	48.30 ± 6.50	46.80 ± 5.90	47.55 ± 6.23	13.10
	High-bolt type	52.50 ± 9.80	50.20 ± 9.20	51.35 ± 9.54	18.58
Total pods per plant	Low-bolt type	203.67 ± 30.20	194.67 ± 28.60	199.67 ± 29.50	14.77
	High-bolt type	267.67 ± 47.50	268.30 ± 51.33	268.03 ± 49.85	18.60
Silique length/cm	Low-bolt type	5.77 ± 0.38	5.83 ± 0.41	5.80 ± 0.40	6.90
	High-bolt type	6.23 ± 0.52	6.07 ± 0.55	6.13 ± 0.54	8.81
Seeds per silique	Low-bolt type	23.67 ± 2.90	24.33 ± 2.40	24.00 ± 2.70	11.25
	High-bolt type	26.33 ± 4.30	25.00 ± 4.50	25.67 ± 4.40	17.14
1000-seed weight/g	Low-bolt type	4.25 ± 0.25	4.32 ± 0.27	4.29 ± 0.26	6.06
	High-bolt type	3.68 ± 0.85	3.73 ± 0.83	3.70 ± 0.84	22.70
Seed yield per plant/g	Low-bolt type	18.60 ± 2.50	17.30 ± 2.20	18.20 ± 2.34	12.86
	High-bolt type	22.80 ± 5.40	21.50 ± 4.90	22.20 ± 5.20	23.42

**Table 6:** Phenotypic traits of elite varieties and control varieties.

Breed Number	Bolting Height (Spring, cm)	Bolting Height (Autumn, cm)	1000-Seed Weight/g	CV/%	Overwintering Rate/%	LT_50_/°C	Comprehensive Score	Ranking
B62	0.00	1.78	4.32	6.06	76.00	−13.76	2.00	1
B74	0.00	21.00	4.29	6.10	75.00	−13.31	1.93	2
B1	0.00	28.33	4.20	6.21	74.00	−12.98	1.88	3
B8	82.00	84.44	3.68	18.58	32.40	−4.51	0.10	93
B75	89.17	90.50	3.70	22.42	32.10	−4.17	0.06	94
B95	94.70	107.50	3.60	22.70	29.40	−4.13	0.00	95

**Table 7:** Primer sequences for RT-qPCR.

Gene	Primer Sequence (5′-3′)
BnaA06g24070D-F	GGGGAAATGGGTTGCTGAGA
BnaA06g24070D-R	AGCTTCTTCCGCGGATTTGA
BnCBF5-F	CCTTCCGCCTCCGATATTCC
BnCBF5-R	TTCCCCAATTTCTCCTCCGC

## Data Availability

The data that have been used in this study are confidential.

## Supplementary Materials

The following supporting information can be downloaded at: https://www.mdpi.com/article/10.3390/plants15091378/s1, Table S1: List of 95 winter-type B. napus accessions used in this study.
