# Supplementary material for: Lower Bolting Height of Winter Rapeseed (Brassica napus L.) Enhances Cold Stress Tolerance and Adaptability to Arid–Frigid Regions in Northern China
Source: Plants (Basel). 2026 Apr 30;15(9):1378. doi: 10.3390/plants15091378 (PMC13164606; doi:10.3390/plants15091378)
Supplement: Supplementary file 1 [file plants-15-01378-s001.zip › plants-4265231-supplementary.pdf]

Table S1. List of 95 winter-type *B. napus* accessions used in this study.

No.	Name	Source	No.	Name	Source
B1	16TS309-4-3	Gansu Agricultural University	B49	2018GL-GAU-53(2020 Cultivar Comparison Trial – Seed Retention)-5	Gansu Agricultural University
B2	16TS309-4-4	Gansu Agricultural University	B50	2018GL-GAU-53(2020 Cultivar Comparison Trial – Seed Retention)-7	Gansu Agricultural University
B3	16NTS309 Regional Trial Ⅲ	Gansu Agricultural University	B51	2019 Low(1)-19(2020 Cultivar Comparison Trial Ⅱ – Seed Retention)	Gansu Agricultural University
B4	16NTS309 Regional Trial Ⅲ-1	Gansu Agricultural University	B52	2019 Low(1)-19(2020 Cultivar Comparison Trial Ⅱ – Seed Retention)-1	Gansu Agricultural University
B5	16NTS309 Regional Trial Ⅲ-2	Gansu Agricultural University	B53	2019 Low(1)-19(2020 Cultivar Comparison Trial Ⅱ – Seed Retention)-2	Gansu Agricultural University
B6	16NTS309 Regional Trial Ⅲ-3	Gansu Agricultural University	B54	2019 Low(1)-19(2020 Cultivar Comparison Trial Ⅱ – Seed Retention)-3	Gansu Agricultural University
B7	16NTS309 Regional Trial Ⅲ-4	Gansu Agricultural University	B55	2019 Low(1)-19(2020 Cultivar Comparison Trial Ⅱ – Seed Retention)-5	Gansu Agricultural University
B8	16NTS309 Regional Trial Ⅲ-5	Gansu Agricultural University	B56	2019 Low(1)-19(2020 Cultivar Comparison Trial Ⅱ – Seed Retention)-6	Gansu Agricultural University
B9	2019QL-GAU-192(2020 Cultivar Comparison Trial Ⅱ – Seed Retention)	Gansu Agricultural University	B57	2019 Low(1)-19(2020 Cultivar Comparison Trial Ⅱ – Seed Retention)-7	Gansu Agricultural University
B10	2019QL-GAU-192(2020 Cultivar Comparison Trial Ⅱ – Seed Retention)-1	Gansu Agricultural University	B58	2019 Low(1)-19(2020 Cultivar Comparison Trial Ⅱ – Seed Retention)-8	Gansu Agricultural University
B11	2019QL-GAU-192(2020 Cultivar Comparison Trial Ⅱ – Seed Retention)-2	Gansu Agricultural University	B59	2019 Low(1)-19(2020 Cultivar Comparison Trial Ⅱ – Seed Retention)-9	Gansu Agricultural University
B12	2019QL-GAU-192(2020 Cultivar Comparison Trial Ⅱ – Seed Retention)-3	Gansu Agricultural University	B60	2019 Low(1)-19(2020 Cultivar Comparison Trial Ⅱ – Seed Retention)-10	Gansu Agricultural University
B13	2019QL-GAU-192(2020 Cultivar Comparison Trial Ⅱ – Seed Retention)-5	Gansu Agricultural University	B61	2019 Low(1)-19(2020 Cultivar Comparison Trial Ⅱ – Seed Retention)-11	Gansu Agricultural University
B14	2019QL-GAU-192(2020 Cultivar Comparison Trial Ⅱ – Seed Retention)-6	Gansu Agricultural University	B62	2018GL-GAU-32-13	Gansu Agricultural University
B15	2019QL-GAU-192(2020 Cultivar Comparison Trial Ⅱ – Seed Retention)-7	Gansu Agricultural University	B63	2019 Low(1)-19(2020 Sampling After Seedling Retention 3)	Gansu Agricultural University
B16	2019QL-GAU-192(2020 Cultivar Comparison Trial Ⅱ – Seed Retention)-8	Gansu Agricultural University	B64	16NTS158-1	Gansu Agricultural University
B17	2019QL-GAU-192(2020 Cultivar Comparison Trial Ⅱ – Seed Retention)-9	Gansu Agricultural University	B65	16NTS158-2	Gansu Agricultural University
B18	2019QL-GAU-192(2020 Cultivar Comparison Trial Ⅱ – Seed Retention)-10	Gansu Agricultural University	B66	16NTS158-4	Gansu Agricultural University
B19	2019QL-GAU-192(2020 Cultivar Comparison Trial Ⅱ – Seed Retention)-11	Gansu Agricultural University	B67	16NTS158-5	Gansu Agricultural University
B20	2019QL-GAU-192(2020 Cultivar Comparison Trial Ⅱ – Seed Retention)-12	Gansu Agricultural University	B68	16NTS158-6	Gansu Agricultural University
B21	2019QL-GAU-201(2020 Cultivar Comparison Trial Ⅲ-4 – Seed Retention)	Gansu Agricultural University	B69	16NTS158-7	Gansu Agricultural University
B22	2019QL-GAU-201(2020 Cultivar Comparison Trial Ⅲ-4 – Seed Retention)-1	Gansu Agricultural University	B70	16NTS158-10	Gansu Agricultural University
B23	2019QL-GAU-201(2020 Cultivar Comparison Trial Ⅲ-4 – Seed Retention)-2	Gansu Agricultural University	B71	16NTS158-11	Gansu Agricultural University
B24	2019QL-GAU-201(2020 Cultivar Comparison Trial Ⅲ-4 – Seed	Gansu Agricultural University	B72	16NTS158-13	Gansu Agricultural University

No.	Name	Source	No.	Name	Source
	Retention)-3				
B25	2019QL-GAU-201(2020 Cultivar Comparison Trial Ⅲ-4 – Seed Retention)-4	Gansu Agricultural University	B73	15NS-45-4 Golden Inbred Line	Gansu Agricultural University
B26	2019QL-GAU-201(2020 Cultivar Comparison Trial Ⅲ-4 – Seed Retention)-6	Gansu Agricultural University	B74	15NS-45-4-7	Gansu Agricultural University
B27	2019QL-GAU-201(2020 Cultivar Comparison Trial Ⅲ-4 – Seed Retention)-7	Gansu Agricultural University	B75	Tianyou 2288	Tianshui Institute of Agricultural Sciences
B28	2019QL-GAU-202(2020 Cultivar Comparison Trial Ⅲ – Seed Retention)	Gansu Agricultural University	B76	Tianyou No.14	Tianshui Institute of Agricultural Sciences
B29	2019QL-GAU-202(2020 Cultivar Comparison Trial Ⅲ – Seed Retention)-2	Gansu Agricultural University	B77	17-2251	Gansu Agricultural University
B30	2018GL-GAU-4(Cultivar Comparison Trial Ⅱ – Seed Retention)	Gansu Agricultural University	B78	16TS312	Gansu Agricultural University
B31	2018GL-GAU-4(2020 Cultivar Comparison Trial Ⅱ – Seed Retention)-1	Gansu Agricultural University	B79	2022SC Gansu Regional Trial Ⅲ Longyou 88	Gansu Agricultural University
B32	2018GL-GAU-4(2020 Cultivar Comparison Trial Ⅱ – Seed Retention)-2	Gansu Agricultural University	B80	Gan Za No.1 Regional Trial Ⅲ	Gansu Agricultural University
B33	2018GL-GAU-4(2020 Cultivar Comparison Trial Ⅱ – Seed Retention)-4	Gansu Agricultural University	B81	Jieyou-1	Gansu Agricultural University
B34	2018GL-GAU-32-4	Gansu Agricultural University	B82	2019 Gansu Winter–Spring Sowing Golden 3-TS312-2	Gansu Agricultural University
B35	2018GL-GAU-32-5	Gansu Agricultural University	B83	2019 Gansu Winter–Spring Sowing Golden Inbred Line 4-TS309-10	Gansu Agricultural University
B36	2018GL-GAU-32-6	Gansu Agricultural University	B84	2022 GAU Research Station Spring Sowing Golden Inbred Line 3-1	Gansu Agricultural University
B37	2018GL-GAU-32-7	Gansu Agricultural University	B85	2022 GAU Research Station Spring Sowing Golden Inbred Line3-2	Gansu Agricultural University
B38	2018GL-GAU-32-8	Gansu Agricultural University	B86	2022 GAU Research Station Spring Sowing Golden Inbred Line3-4	Gansu Agricultural University
B39	2018GL-GAU-32-9	Gansu Agricultural University	B87	2019 Gansu Winter–Spring Sowing Golden 3-1	Gansu Agricultural University
B40	2018GL-GAU-32-10	Gansu Agricultural University	B88	2019 Gansu Winter–Spring Sowing Golden 3-2	Gansu Agricultural University
B41	2018GL-GAU-32-11	Gansu Agricultural University	B89	2019 Gansu Winter–Spring Sowing Golden 3-2 Bagged Selfing Line	Gansu Agricultural University
B42	2018GL-GAU-32-12	Gansu Agricultural University	B90	2022GAU-2019 Gansu Winter–Spring Sowing Golden 3-Open Pollination Line	Gansu Agricultural University
B43	2019 Low(1)-19(2020 Sampling After Seedling Retention 1)	Gansu Agricultural University	B91	2022GAU-2019 Gansu Winter–Spring Sowing Golden 3 Bagged Selfing Line	Gansu Agricultural University
B44	2018GL-GAU-53	Gansu Agricultural University	B92	2022GAU-2019 Gansu Winter–Spring Sowing Golden Inbred Line 3-7	Gansu Agricultural University
B45	2018GL-GAU-53(2020 Cultivar Comparison Trial – Seed Retention)-1	Gansu Agricultural University	B93	2022GAU-2019 Gansu Winter–Spring Sowing Golden Inbred Line 3-8	Gansu Agricultural University
B46	2018GL-GAU-53(2020 Cultivar Comparison Trial – Seed Retention)-2	Gansu Agricultural University	B94	2022GAU-2019 Gansu Winter–Spring Sowing Golden Inbred Line 3-9	Gansu Agricultural University
B47	2018GL-GAU-53(2020 Cultivar Comparison Trial – Seed Retention)-3	Gansu Agricultural University	B95	Tianyou 608	Tianshui Institute of Agricultural Sciences
B48	2018GL-GAU-53(2020 Cultivar Comparison Trial – Seed Retention)-4	Gansu Agricultural University			
